# The Book World of Medicine and Science

**Published:** 1911-09-09

**Authors:** 


### The Book World of Medicine and Science.

The Medical Aspects of the New Edition of the Encyclopædia Britannica, published by the Cambridge University Press.

Within the limits of a brief review it is impossible to criticise in detail the numerous articles of medical and surgical importance in the new edition of the Encyclopædia Britannica. All that the most conscientious reviewer can do is to read selections from these articles, and thereby form a judgment on the whole. First of all it may be stated that the selection of topics is in the main very careful and rational, and that their relative importance is fairly accurately reflected in the amount of space afforded to each. In other words, the editing of the articles has been carefully and satisfactorily performed. There are, it is true, some articles whose authors have been allowed to run riot rather, and to dilate upon their themes at perhaps too great length. The valuable article on the Brain, for instance, which is signed with Professor Sherrington's initials, is so lengthy and technical as almost to be a text-book in itself. We venture the opinion that in this case and in one or two others compression might usefully have been resorted to. Next it is to be recorded with satisfaction that no attempt is made to render the articles a *vade mecum* of diagnosis and self-treatment for the general public. It is true that a good deal of information is given concerning the lines of treatment, the prognosis, diagnosis, and pathology of many diseases; but the facts are stated in such a way as to discourage readers from attempting the dangerous paths of self-drugging, and to encourage their reliance on a practitioner. Routine methods of treatment are frequently warned against, very properly, and the importance of rest is duly insisted on in many of the articles. In the articles on surgical subjects, which are numerous, especial attention may be directed to those signed with the initials of Mr. Edmund Owen. They are always sane and practical, full of surgical commonsense. Whether they have been waiting some months or years for publication we do not know; but certainly some of the views expressed are, to say the least of it, conservative. To give an instance, the article on hernia would leave an intelligent lay reader with the impression that the value of the operation of radical cure is still in dispute, and would encourage him to prefer truss treatment for a simple reducible hernia. Possibly Mr. Owen still holds these opinions about radical cure, but we doubt if he would be allowed to pass the Fellowship of the College nowadays if he expressed them to the examiners. The article on homœopathy is interesting, especially when compared with the late Dr. Glover's terrific onslaught on this creed in the last edition of the Encyclopædia. Dr. Glover wrote a tirade of concentrated invective which was a masterpiece of English composition and of destructive ridicule. This is now replaced by a much more colourless, though perhaps fairer, account evidently penned by someone who sympathises with the doctrines of Hahnemann. As a matter of fact, this new article really glosses over the inconsistencies and illogicalities of homœopathy and the trickery which has been so frequently associated with it; and errs as much on the side of benevolence towards the ill-balanced founder of the cult as the previous article did in intolerant sarcasm. There are also several articles on historical aspects of medicine, which are interesting and valuable; to take an instance, the biography of John Hunter may be mentioned, while the account of the development of the science of anatomy is excellent. The articles on mental disease are particularly full and conspicuously well arranged: they are not all by the same author, apparently, but probably have been coordinated by some expert in this branch of practice. Some of the medical articles, like some of those on surgical subjects, appear to have been written some years, though they have not thereby been allowed to fall out of date. Under the heading gynæcology appears a condensed abstract of the principal diseases dealt with by specialists in that science; this would have been far better omitted, as it is useless to medical men, and merely terrifying to those lay readers whose tastes are sufficiently morbid to induce them to read it. The author of the brief article on anæsthetics has achieved considerable proficiency in the mis-spelling of proper names. Lastly, it must be confessed that the illustrations are for the most part extremely crude, and that many of them would be much better omitted. In general, however, notwithstanding these few criticisms on minor points, it is only fair to say that the medical sections of this Encyclopædia are extraordinarily good, and that they are worthy of the confidence which the public has learned to bestow on everything that appears in this work.

The Surgery of the Diseases of the Appendix Vermiformis and their Complications. By W. H. Battle, F.R.C.S., and E. M. Corner, M.D., M.C., F.R.C.S. Second edition. 1910. (London: Constable and Co., 10 Orange Street, Leicester Square, W.C. 10s. 6d. net.)

This book needs little introduction to the practitioner, for its popularity is already assured. It is undoubtedly one of the best monographs on the various lesions to which the human appendix is subject. Since the first edition was published the literature on the appendix has increased to an awe-inspiring extent, so that it is quite impossible for the average practitioner to keep pace with it. Here however we have the best of it condensed and elucidated. The task that the writers had before them was not a small one, but they have grappled well with the wealth of material, and the result is surprisingly satisfactory. The chapter on the complications of appendicitis must remain the most important, and it is very clearly written so that the reader is left in no doubt as to what these complications mean. The indications for operative treatment are fully discussed, the operations are described in detail, and the rarer lesions (for example, carcinoma, the chapter on which is an admirable summary of the literature) are adequately dealt with. A feature of the book is the relatively large amount of space devoted to the really important subjects—those that are of primary interest to the practitioner. The subject of appendicitis in relation to life insurance is considered in the concluding chapter, and a large number of illustrations, which are almost without exception elucidative, have been added. The authors state that they have omitted details of cases so as not to obscure the more important matter; this we regret, since in our opinion illustrative cases add to the usefulness of a book which is admittedly a practitioner's manual. We usually refrain from commenting on the manner in which a book is written, on its style and literary finish. In this case however the usefulness of the work is seriously hampered by the slovenly way in which the subject has been presented to the reader. There are many printer's errors, but a much more glaring defect is the peculiar method of punctuation and the equally peculiar elliptic construction which the authors have adopted, both of which tend at times to obscure their meaning. Two instances will suffice, both on the same page (p. 68) and both apparently complete sentences. "But the considerations are such that an opinion may be formed either one way or the other, or else none can be." "If operation is called for after the third day, it cannot but be felt that the case would have been far better treated had it been so submitted earlier." Both these sentences need some mental effort to be understood, and they are not singular instances. The authors should carefully revise the next edition and submit the proof-sheets to some literary friend for criticism.

The Practice of Medicine. By Frederick Taylor, M.D., F.R.C.P. (London: J. and A. Churchill, 7 Great Marlborough Street. 1911. Price 18s. net.)

This, the ninth edition of a deservedly popular textbook, may justly claim to be the most comprehensive and up-to-date manual of medicine for the use of English students. The admirable way in which every subject has been treated, by an arrangement which, while it secures due condensation, omits nothing that is of material importance either from a practical or from an examination point of view, makes the book more readable than such abridged "monographic" textbooks usually are. Some of the chapters, indeed, notably those on infectious diseases and on diseases of the nervous system, remind one of the author's excellent style and literary ability, which are displayed in his article on Myelitis in Allbutt's System. Naturally the book has increased in size and weight, for many new paragraphs have been added, among the additions being notes on pellagra, dilatation of the colon, lymphatism, Oppenheim's disease, meralgia paræsthetica, enterospasm, and congenital family cholæmia. The old sections have been brought up to date and, taken as a whole, the book is a great advance upon the last edition. Many new illustrations have been added, some of which greatly increase the interest of the book; a few, on the contrary, are by no means helpful; thus the most expert pathologist would be at a loss to diagnose fig. 33, which is stated to be the spinal cord, showing the cavitation in syringomyelia, but which has an unholy resemblance to the propeller of a biplane. We must admit, too, that we fail to see the usefulness of some of the *x*-ray photographs showing thoracic lesions. The book is obviously intended for students, and unless the student is shown a normal *x*-ray photograph of the heart he will be utterly unable to distinguish the slightest abnormality in Plate VI. (page 604), which shows a not very marked "round heart" in a case of mitral stenosis. A little more attention ought to have been given to foreign work in many subjects; for example, in the chapter dealing with diseases of the intestine, the question of constipation is discussed, and reference is made to the work of a Guy's student, but the earlier and more important work done in America and in Germany is not even mentioned.

We have no desire to criticise Dr. Taylor's work in detail, and the only cavilling criticism possible must be directed to details, for the whole is so good that there can be nothing but praise for it. The volume exactly meets a want—to use a hackneyed and ungrammatical expression, a long-felt want even. It is an admirable handbook for the ward clerk who is commencing his clinical work, and is indispensable for the student who is preparing for his examinations in medicine. The practitioner will find in the generally too short sections dealing with treatment many very useful hints which should make the book valuable to him as well. But it is pre-eminently a student's handbook, and as such perhaps the best on medicine that is to be procured in the English language.

Faith, Medicine, and the Mind. By Charles Reinhardt, M.D., D.S. Brux., L.S.A. (London: The London Publicity Co., Ltd. 1911. Pp. 281 and vi. Price, 5s. net.)

Dr. Reinhardt feels that a rational explanation of all the phenomena of faith-healing is available. With that end in view he has drawn upon his own experiences in the occult, which date from the time he was a medical student. His accounts of his early experiments and experiences in hypnotism cannot fail to fascinate both the lay and the scientific reader. Both will sympathise with him in his terrifying experience with a subject who when under hypnotic influence failed to respond to the suggestion that he should wake up. How the peril was avoided we must leave it to the reader of the book to find out for himself. The author has of set purpose avoided touching on subjects of religious opinion, contenting himself with endeavouring to throw some light upon the psychology of the faith that heals. The question as to whether he has done so or not can, we think, be answered in the affirmative, though no doubt some will be found to disagree with this conclusion. Nevertheless, whatever be the opinion of different readers, it cannot be denied that Dr. Reinhardt has produced a most interesting and readable volume on a subject which is to-day receiving a great deal of attention, and which deserves the consideration of all medical men who wish to combat the insidious machinations of the army of quacks of all kinds who are making capital out of the interest taken by a gullible public in matters pertaining to the occult.

Phases of Evolution and Heredity. By David Berry Hart, M.D., F.R.C.P.E. (London: Rebman, Limited. 1910. Pp. 259. Price 5s. net.)

This short work should serve as an introduction for the student to the study of the various theories connected with the science of evolution and heredity. At the same time the language in which it is written is simple enough to be understood by the average layman with no anterior acquaintance with the subject. The first part of the volume deals with the various "isms" connected with the names of Darwin, Weismann and Mendel, and that which may be considered unsatisfactory in each is pointed out. Brief mention is made of biometry of the Galton-Pearson school, and of Richard Semon's mnemism. An intrinsic theory of variation and its transmission is next discussed, after which follow chapters on mutation, heredity, and heredity in disease. Next there are touched upon evolution and controversy, the handicap of sex, and evolution in religion. The book is brought to a close by an interesting chapter on "Men who have Revealed Themselves." Of these there are but three great ones—Rousseau, Pepys, and Amiel. They represent three absolutely distinct types—Rousseau the frankly brutal sensualist; Pepys the *bon-viveur*, the "cleaner-sinner" whose conduct amuses us while we deplore it; and Amiel the "pure-minded," the saint, so to speak, of the trio. In attempting to find a place in Evolution for these three exceptions to the general personal reticence of mankind, the author makes use of an idea of Taine, who speaks of Napoleon as one of the Medici come to life. On this analogy Dr. Hart considers Pepys a reincarnation of the old Roman consuls, and Amiel a Buddhist in his nature. He makes no attempt to dispose of Rousseau; but then, as he says, "Heredity has its laws, of which we know little yet; but the one we know least of is that regulating the appearance of men of genius, or of such belated dwellers on this planet as Pepys and Amiel, the literary atavists of Evolution."

